# Proliferation ability of particulated juvenile allograft cartilage

**DOI:** 10.1186/s13018-020-02199-z

**Published:** 2021-01-14

**Authors:** Changgui Zhang, Xingyu Zhao, Yunong Ao, Jin Cao, Liu Yang, Xiaojun Duan

**Affiliations:** Center for Joint Surgery, Southwest Hospital, Third Military Medical University (Army Medical University), Chongqing, 400038 China

**Keywords:** Particulated juvenile allograft cartilage, Autologous cartilage chip transplantation, Donor cells, Ki-67, Lin28, Proliferation

## Abstract

**Background:**

Particulated juvenile allograft cartilage (PJAC) has a good short-term clinical efficacy in repairing articular cartilage defects, but the proliferation ability of PJAC and the biological characteristics of transplanted cells after transplantation are still unclear.

**Purpose:**

To study the cartilage proliferation ability of PJAC in repairing full-thickness cartilage defects and the reasons for proliferation to provide experimental evidence for its clinical application.

**Study design:**

Controlled laboratory study.

**Methods:**

Twenty Guizhou minipigs were randomly divided into the experimental group and control group. In all minipigs, an 8-mm cylindrical full-thickness cartilage defect was created in the femoral trochlea of one knee. The experimental group received PJAC transplantation from five juvenile donors of Guizhou minipigs (PJAC group; *n* = 10) and the control group received transplantation of autologous cartilage chips (ACC group; *n* = 10). Both groups were followed at 1 and 3 months after surgery, immunohistochemical evaluation of the tissue sections Ki-67 and Lin28 was conducted, the positive rate was calculated according to the staining, and the proliferation ability of PJAC was analyzed.

**Results:**

All 20 Guizhou minipigs were followed, and there was no infection or incision healing disorder after surgery. By Ki-67 and Lin28 immunohistochemical tests, the positive rate of Ki-67 was 88.9 ± 0.2% in the PJAC group and 28.3 ± 3.6% in the ACC group at 1 month, and the difference was statistically significant (*P* < 0.05); the positive rate of Lin28 was 34.6 ± 3.3% in the PJAC group and 7.6 ± 1.4% in the ACC group at 1 month, and the difference was statistically significant (*P* < 0.05). At 3 months, the positive rates of Ki-67 in the PJAC group and ACC group were 53.6 ± 6.9% and 1.97 ± 0.3%, respectively (*P* < 0.05); the positive rates of Lin28 were 86.6 ± 3.3% and 1.4 ± 0.3%, respectively (*P* < 0.01).

**Conclusion:**

A large animal model was established with Guizhou minipigs, and the expressions of Ki-67 protein and Lin28 protein detected by immunohistochemistry in the repaired transplanted tissue of the PJAC group were stronger than those of adult cartilage. The proliferation of PJAC within 3 months of transplantation was stronger than that of adult cartilage. The enhanced expression of Lin28 may be one of the mechanisms by which PJAC achieved stronger proliferation ability than adult cartilage. PJAC technology has shown good application prospects for repairing cartilage defects.

## Background

Articular cartilage is often damaged due to traumatic and degenerative factors, resulting in cartilage defects. Meanwhile, due to the lack of blood supply of articular cartilage, the proliferation ability of adult chondrocytes is extremely low [1, 2]. The current treatment methods have many disadvantages. For instance, the long-term efficacy of microfractures is not satisfactory; autologous cartilage chip (ACC) also has limitations due to the limited proliferation potential of adult cartilage [3–6]. In recent years, studies have found that juvenile chondrocytes have strong proliferation potential [7, 8]. Namba et al. found in the injury model of fetal sheep that the articular cartilage defects they created were completely and spontaneously repaired after 28 days [7]. Bonasia et al. found through in vitro cell culture that the proliferation of juvenile chondrocytes was significantly better than that of adult chondrocytes [8]. Based on the characteristics of strong proliferation of transplanted juvenile cartilage, particulated juvenile allograft cartilage (PJAC) from donors under 13 years old was prepared and stored at low temperature, and then applied with fibrin glue to the cartilage defects [9]. At present, DeNovo NT, a commercial product of PJAC, has been widely used in clinical practice and has achieved satisfactory therapeutic effects [10–15]. Compared with microfractures and ACC, PJAC transplantation has better long-term efficacy. The patient’s clinical symptoms have improved significantly, and the imaging data have shown better results, bringing new hope for the treatment of cartilage damage.

Although the repair effect of PJAC on cartilage defects is good, the proliferation ability of chondrocytes after transplantation and the related mechanisms of repair are still unclear. In our previous study, we used Guizhou minipigs to establish large animal models and compared the repair effect between the PJAC and ACC techniques; the results showed that there was no significant difference between the two techniques at 6 months after transplantation. This indicated that as time went by, PJAC could demonstrate better and better repair effect. In this study, we proved that PJAC could be used to treat cartilage defects and had good repair effect. We simulated the clinical cartilage defect repairing process of PJAC through large animal experiments, which provided an experimental basis for its clinical application [16, 17]. Although there were studies on juvenile cartilage by in vitro cell culture showing its proliferation ability was stronger than that of adult one, there has been no large animal experiment to investigate the proliferation ability of juvenile cartilage in vivo. Ki-67 is a nuclear antigen, which exists in proliferating cells, but not in quiescent cells. The immunostaining of Ki-67 is often used to determine the proliferation ability of cells in a tissue [18–20]. It can not only determine the proliferation ability of tumor cells, but also the proliferation ability of chondrocytes [21, 22]. Moreover, the reason why the proliferation ability of juvenile cartilage is stronger than that of adult one is still unclear. Some studies found that the key gene controlling the proliferation of juvenile cartilage was the protein coding gene Lin28. We hypothesize that the repair and proliferation ability of juvenile cartilage may be due to the activation of Lin28, which combines with several metabolic enzymes, enhances its function, and promotes oxidative metabolism and the bioenergy characteristics of chondrocytes to facilitate the repair and proliferation of juvenile cartilage [23].

This study aims to use immunohistochemical methods to study the proliferation ability of juvenile cartilage after PJACs are transplanted into cartilage defects, to explore the reasons for the strong proliferation ability of transplanted PJAC, to prove it is stronger in proliferation ability than adult cartilage, and to lay a solid theoretical foundation for elucidating the pathway mechanism of PJAC in repairing articular cartilage defects in the future.

## Materials and methods

### Animal preparation

We chose five male juvenile Guizhou minipigs younger than 2 months, weighing 5.1–6.0 kg and aged 1.0–1.3 months, as the donors of PJAC. Twenty female mature and healthy adult Guizhou minipigs, weighing 28.4–34.5 kg and aged 18–20 months, were used for the creation of cartilage defect model. Ten of the adult minipigs received PJAC transplantation (PJAC group) and 10 received ACC transplantation (ACC group). All animals in this study were provided by the Large Animal Experimental Center of the Army Medical University and were raised according to the Experimental Animal Care Specifications. This study was approved by the Experimental Animal Welfare Ethics Committee of Army Medical University (No. AMUWEC2017147).

### Surgical procedures

#### PJAC preparation

The experiment was carried out in five batches. In the PJAC group, one juvenile minipig and two adult minipigs were used in each batch. The juvenile minipig was intravenously injected with sodium pentobarbital (3%, 1 mL/kg; Huamu Pharmaceutical Co., Ltd., China) and euthanized after anesthesia. The knee of the male minipig was cut aseptically; the articular cartilage was obtained from the articular surface of distal femur, and PJAC cubes of about 1 mm^3^ were cut with a scalpel and stored aseptically in a sterile test tube, mixed with a small amount of saline to keep the particulated cartilage moist, and then stored in a refrigerator at 4 °C. The pre-preparation of fibrin glue is as follows: fibrinogen (500 mL, 20 mg/mL; Sigma), CaCl_2_ (20 mL, 50 mmol/L; Solarbio), and thrombin (500 mL, 250 m/mL; Sigma) were separately put into 3 aseptic centrifuge tubes; cartilage chip-fibrin glue was formulated with PJAC and the 3 reagents before transplantation.

#### Cartilage defect creation

The adult female minipigs received intramuscular injection of xylazine (0.1 mL/kg; Huamu Pharmaceutical Co., Ltd., China) and atropine (1.5 mL/each minipig; Huamu Pharmaceutical Co., Ltd., China) to induce muscle relaxation, followed by intravenous injection of sodium pentobarbital (3%, 1 mL/kg; Huamu Pharmaceutical Co., Ltd., China) and fentanyl (0.15 mL/kg/h; Janssen Pharmaceutica, Belgium) for anesthesia. The surgery was performed after the anesthesia took effect. All surgeries were performed by the same senior surgeon. One lower extremity was randomly selected for surgery. Incision was made on the medial epidermis of the knee joint and went deeper layer by layer to reach the joint capsule; the joint capsule and medial patellar retinaculum were cut off to dislocate the patella outward and expose the femoral trochlea. A trephine (8 mm in diameter) was applied to create a defect perpendicular to the knee joint surface without destroying the subchondral bone. A curette was used to debride the cartilage defect. The defect area was then rinsed with normal saline to ensure no cartilage debris would remain.

#### PJAC and ACC transplantation

In the PJAC group, cartilage chip-fibrin glue formulated by the freshly prepared PJAC obtained on the day of surgery and the above-mentioned pre-packed reagents were implanted into the cartilage defect area. After about 5 min, the patella was restored to perform 45-degree flexion and extension activities; then, the patella was pushed aside to check whether the transplanted PJAC was fixed to the defect area with the gel. Absorbable sutures were applied layer by layer. In the ACC group, 1 mm^3^ of ACC cubes prepared from the cartilage defect model were transplanted back to the defect area. After awakening from anesthesia, the minipigs were placed in the Large Animal Experimental Center and given formula feed once a day (0.5 kg, Tianzheng Company, China). They were able to move freely without immobilization. Penicillin (0.03 mL/kg, Harbin Pharmaceutical, China) was injected into each minipig intramuscularly, twice a day for 1 week.

#### Sample collection and processing

Follow-ups were carried out at 1 and 3 months after surgery. At each follow-up point, 5 Guizhou minipigs were euthanized in each group and 5 samples were collected for processing. The collected samples were fixed and decalcified with ethylenediaminetetraacetic acid (EDTA) solution for 2 weeks. After embedding in paraffin, the samples were cut into sections with a thickness of about 5 μm, and 5 sections were selected in each sample to be used in the subsequent immunohistochemical tests.

#### Immunohistochemical tests

The sections were deparaffinized and hydrated, repaired by citric acid (pH 6.0) with microwave, cooled at room temperature, treated with 3% hydrogen peroxide for 25 min to block endogenous peroxidase, washed with PBS, marked a circle with an immunostaining pen, sealed with 3% bovine serum albumin (BSA) at room temperature for 30 min, added with primary antibodies (anti-Ki-67, ab15580, 1:200, Abcam; anti-Lin28, ab63740, 1:50, Abcam), stored in refrigerator at 4 °C overnight, washed with PBS, added with secondary antibody of goat anti-rabbit IgG-HRP (GB23303, 1:200), incubated for 50 min at room temperature, washed with phosphate buffer saline (PBS), and then added with diaminobenzidine (DAB) chromogenic reagent (G1212-200, 1:5000) under the microscope. If they showed obvious positive signal and clean background, the developing reaction was terminated with pure water. The sections were counterstained with hematoxylin, differentiated with hydrochloric acid, and stained back to blue by ammonium hydroxide; then, they were dehydrated and mounted with resin. Images were collected under the microscope.

### Statistical analysis

The positive rate of immunohistochemistry was analyzed according to tissue staining tests, and statistical analysis was performed by SPSS 23.0. Measurement data were expressed as mean ± standard error of mean (SEM) to analyze whether the data conformed to the normal distribution. Paired *t* test was used for statistical analysis; *P* < 0.05 was considered as statistically significant.

## Results

### Immunohistochemical results

**Fig. 1:**
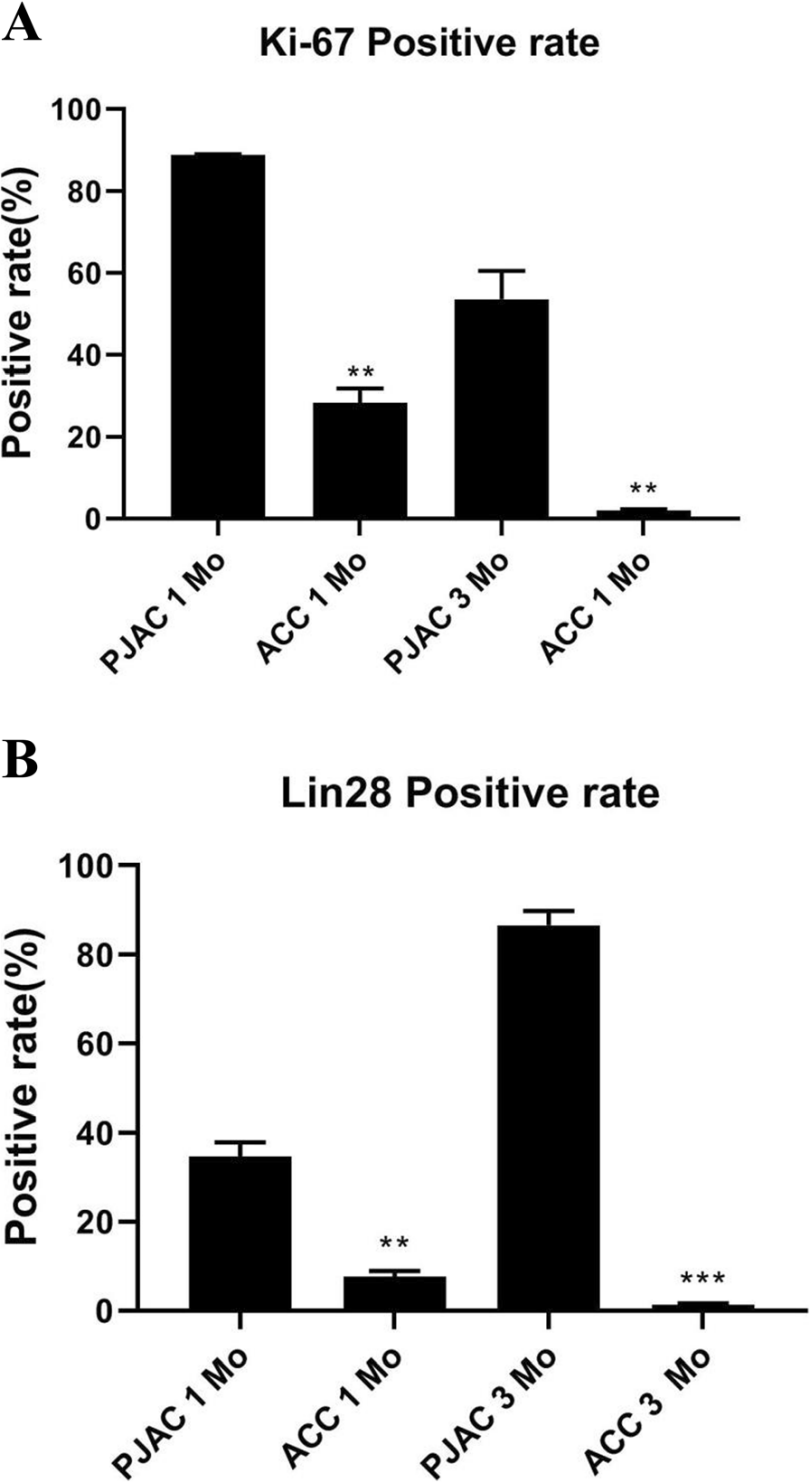
**a** Statistics chart of Ki-67 positive rate. **P < 0.05 indicates a statistically significant difference. PJAC, particulated juvenile allograft cartilage; ACC, autologous cartilage chip. **b** Statistics chart of Lin28 positive rate. **P < 0.05 indicates a statistically significant difference. ***P < 0.01 indicates a statistical significance

**Fig. 2:**
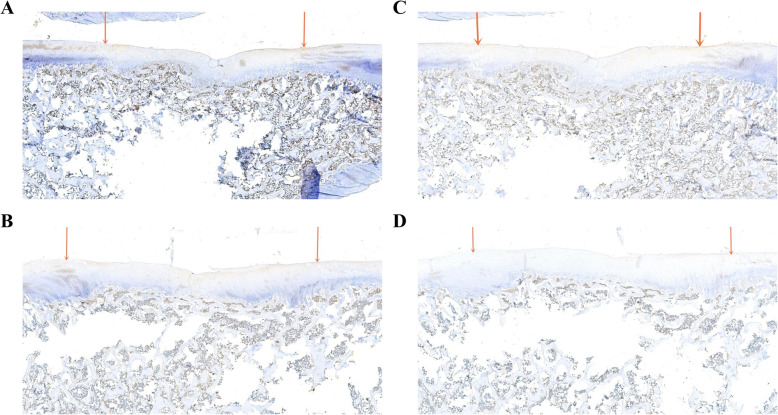
**a** Immunohistochemistry of Ki-67 in the PJAC group at 3 months (× 26 full scan). The area between the red arrows is the transplanted zone. **b** Immunohistochemistry of Ki-67 in the ACC group at 3 months (× 22 full scan). The area between the red arrows is the transplanted zone. **c** Immunohistochemistry of Lin28 in the PJAC group at 3 months (× 22 full scan). The area between the red arrows is the transplanted zone. **d** Immunohistochemistry of Lin28 in the ACC group at 3 months (× 22 full scan). The area between the red arrows is the transplanted zone

**Fig. 3:**
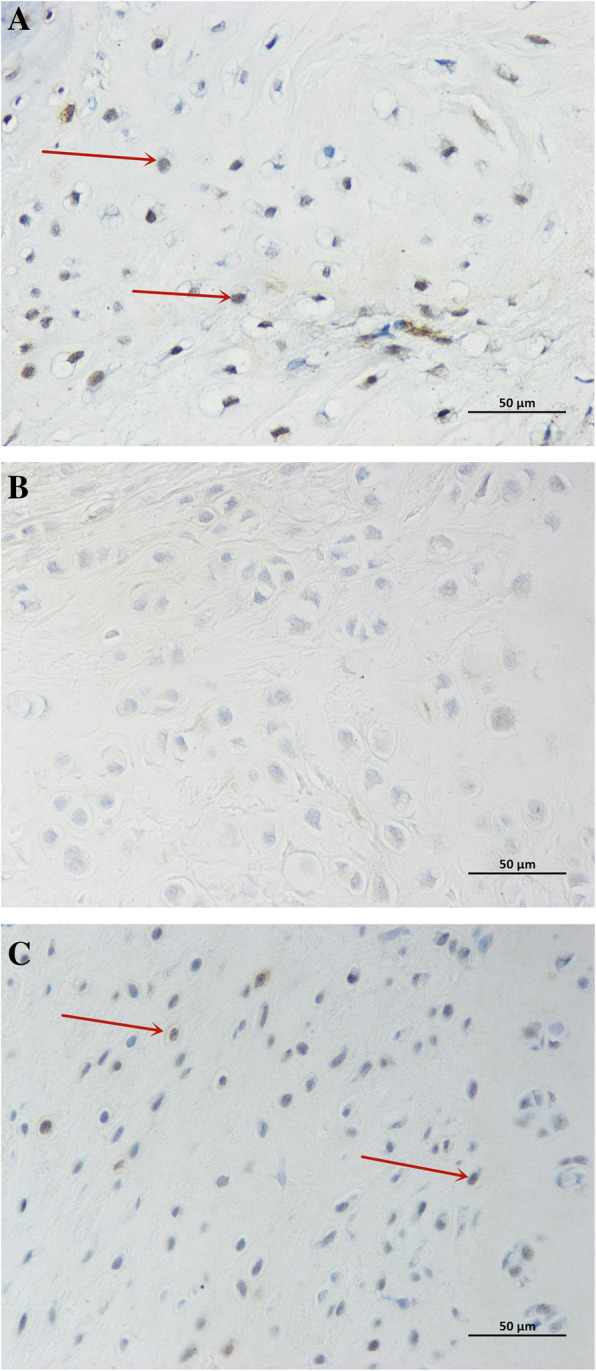
**a** Immunohistochemistry of Ki-67 in the PJAC group at 1 month (× 400 light microscope). The red arrow indicates positive cells. **b** Immunohistochemistry of Ki-67 in the ACC group at 1 month (× 400 light microscope). **c** Immunohistochemistry of Ki-67 in the PJAC group at 3 months (× 400 light microscope). The red arrow indicates positive cells. **b** Immunohistochemistry of Ki-67 in the ACC group at 3 months (× 400 light microscope)

**Fig. 4:**
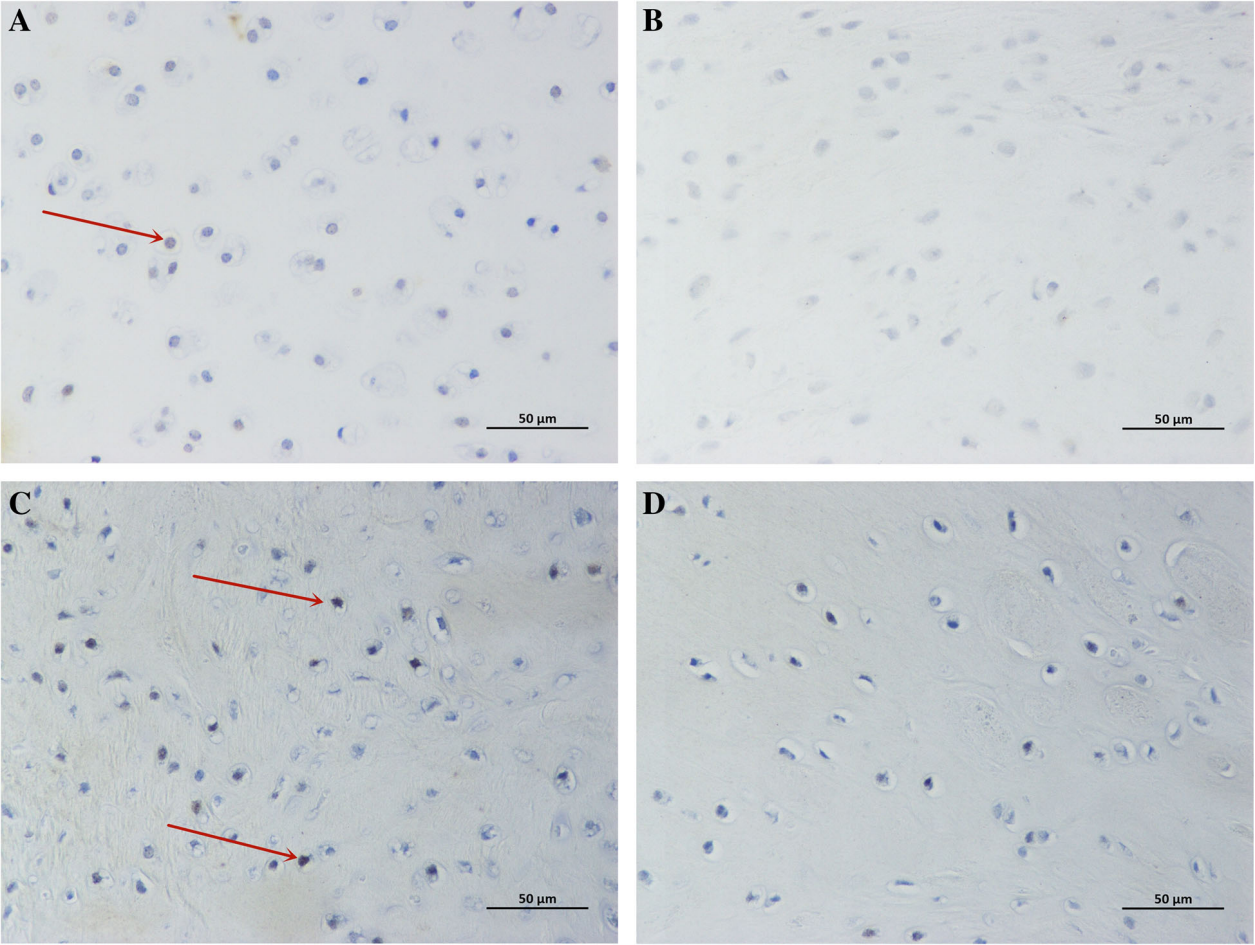
**a** Immunohistochemistry of Lin28 in the PJAC group at 1 month (× 400 light microscope). The red arrow indicates positive cells. **b** Immunohistochemistry of Lin28 in the ACC group at 1 month (× 400 light microscope). **c** Immunohistochemistry of Lin28 in the PJAC group at 3 months (× 400 light microscope). The red arrow indicates positive cells. **d** Immunohistochemistry of Lin28 in the ACC group at 3 months (× 400 light microscope)

We performed immunohistochemical tests on the Ki-67 and Lin28 of samples at 1 and 3 months and found positive cells in both groups. To describe the changes in immunostaining, we extracted 3 regenerated tissue areas from different samples; Image-Pro Plus 6.0 (Media Cybernetics, Inc., Rockville, MD, USA) was applied to conduct semi-quantitative analysis to obtain the number of positive cells and the total number of cells and to calculate the percentage of positive cells. At 1 month postoperatively, the positive rates of Ki-67 in the PJAC group and ACC group were 88.9 ± 0.2% and 28.3 ± 3.6%, respectively, and the difference was statistically significant (*P*< 0.05); the positive rates of Lin28 in the two groups were 34.6 ± 3.3% and 7.6 ± 1.4%, respectively (*P* < 0.05). At 3 months, the positive rates of Ki-67 in the PJAC group and ACC group were 53.6 ± 6.9% and 1.97 ± 0.3%, respectively (*P*< 0.05); the positive rates of Lin28 in the two groups were 86.6 ± 3.3% and 1.4 ± 0.3% , respectively (*P* < 0.01) (Figs. 1, 2, 3, and 4).

## Discussion

This study aims to compare the repair effect of PJAC and ACC. It is the first large animal experiment that has proven that the juvenile cartilage has stronger proliferation ability than the adult one and that the juvenile cartilage has stronger proliferation potential. It also proposes that the proliferation ability of juvenile cartilage may be related to the activation of Lin28. Lin 28 could combine with several metabolic enzymes to enhance its functions and promote oxidative metabolism (glycolysis and oxidative phosphorylation) and the bioenergy characteristics of chondrocytes to facilitate the repair and proliferation of juvenile cartilage. We hope that this study can provide some reference for future clinical application of PJAC technology. At present, PJAC has been proven to be one of the safest and most effective treatments for focal articular cartilage defects. Compared with ACC transplantation that can only produce better results in younger people, PJAC has been successfully applied in all age groups with high-quality repaired tissues. In 2013, Tompkins et al. applied PJAC to treat 16 patients with patellar cartilage lesions; they found that at 28 months postoperatively, not only the International Knee Documentation Committee (IKDC) scores of the patients were significantly improved, but also 89% and 73% of their MRI results showed that the defect area appeared to be “normal or near-normal” [24]. In 2014, Farr et al. followed 25 patients after PJAC surgery for 24 months; they found that the scores of IKDC, Knee Injury and Osteoarthritis Outcome Score (KOOS), and visual analog scale (VAS) were all improved, and biopsy of the repair tissue of 8 patients showed that the tissue was composed of hyaline change and fibrous cartilage [11]. The success of PJAC surgery is based on the proliferation ability of juvenile cartilage in vitro [7, 8]. Previous in vitro studies have proven that juvenile cartilage has a strong proliferation ability compared with adult cartilage. This may be the reason why PJAC’s clinical efficacy is better than ACC. The most important limiting factor of ACC is the low proliferation ability of adult cartilage [3–6], leading to poor prognosis of clinical symptoms and imaging data, and unstable repaired tissue.

We used immunohistochemistry to analyze the cartilage proliferation through antigen and antibody reactions. By analyzing the Ki-67 positive rate, we found an interesting phenomenon: the expression of Ki-67 in the PJAC group was stronger than that of ACC group within 3 months. This may be the reason why the repair ability and proliferation ability of juvenile cartilage continue to increase. Recent studies have shown that Ki-67 protein was originally defined by the prototype monoclonal antibody Ki-67 (Gerdes et al. 1983) [25], which was produced by immunizing mice with the nucleus of Hodgkin’s lymphoma cell line L428. The name is derived from the city of origin and the number of original clones on 96-well plates. Since the antigen was not identified initially, it was mainly called the Ki-67 antigen. When the antigen was identified to be a protein whose primary structure could be inferred from the corresponding cDNA, it was found that it had no homology with any known polypeptide. The prototype antibody and antigen are called Ki-67 antibody and Ki-67 protein, respectively. Ki-67 protein is expressed in the G1, G2, S, and M cycles of active cell proliferation, but not in the resting G0 cell phase. Based on this feature, immunostaining of Ki-67 is often used to determine the ability of cell proliferation in a tissue, not only in the field of tumor cell proliferation, but also the proliferation ability of cartilage [18–22]. This study investigated the expression of proliferation-related antigen Ki-67 in transplanted cartilage and showed the immunohistochemical positive rate of juvenile cartilage was more than that of adult cartilage. This result proved that the proliferation ability of juvenile cartilage was stronger than that of adult cartilage. Studies have found that Lin28 is a highly conserved RNA-binding protein that is expressed during embryogenesis and plays a role in growth, pluripotency, and metabolism. In order to determine whether Lin28 affects adult tissue repair, researchers have used several injured tissue models to reactivate the expression of Lin28. They found that Lin28 combined with several metabolic enzymes and enhanced its function and promoted oxidative metabolism (glycolysis and oxidative phosphorylation) and the bioenergy characteristics of embryonic cells to facilitate tissue repair. Finally, it was found that the re-expression of Lin28 promoted the repair and regeneration of the cartilage, bone, and interstitial tissue after ear and finger injuries. Lin28 may be the key gene that makes the proliferation ability of juvenile cartilage stronger than that of the adult one. In this study, we further verified this hypothesis and found through large animal experiments that the Lin28 protein expression of juvenile chondrocytes was stronger than that of adult ones, which might have a strong correlation with the proliferation ability of juvenile cartilage. The mechanism of Lin28 facilitating tissue repair and proliferation of juvenile cartilage needs further research [23].

Recent studies have found that PJAC surgery is very effective in clinical application, and the clinical symptoms and imaging data of patients have improved significantly. However, there has been no animal study to verify whether the proliferation ability of juvenile cartilage is stronger than the adult one. The purpose of our large animal experiment is to confirm the proliferation ability and potential of PJAC by immunohistochemical technique to promote its further application. The results of this study are similar to previous studies. Goss et al. created oral mucosal, skin, and cartilage wounds of the fetus in the rat’s uterus and observed the wounds within 72 h; the cartilage wounds healed rapidly without inflammation during the healing process [26]. Cherukupally et al. studied the staining of proliferation markers after cricoid cartilage injury in rabbits of 1.5, 4, and 8 weeks; they found that the proliferation markers were progressively attenuated with age [27]. Wagner et al. found that compared with adult cartilage, neonatal rat cartilage had the ability to rapidly regenerate without scarring after full-thickness incision [28]. Namba et al. found that in the injury model of fetal sheep, the partial-thickness defect of articular cartilage was completely and spontaneously repaired after 28 days [7]. By culturing juvenile cartilage and adult cells in vitro, Bonasia et al. found that the proliferation of juvenile cartilage was significantly better than that of adult cells and mixed-cultured cells, while there was no significant difference in the proliferation of mixed-cultured cells and adult cells [8]. Marmotti et al. combined the juvenile cartilage particles and mature cartilage particles with hyaluronic acid scaffolds cultured in vitro and found that the proliferation ability of juvenile cartilage was significantly stronger than that of mature cartilage, and the regeneration ability would decrease with age [29]. These studies have confirmed the strong proliferation ability of fetal and juvenile cartilage. To our knowledge, this study is the first to have proven this phenomenon in large animals. We hope it can provide a scientific basis for better therapeutic effects in the clinical application of PJAC.

Adult cartilage has very weak proliferation ability due to few blood vessels and lymphoid tissue. According to previous studies, for adult human, cartilage defects larger than 10 mm cannot be self-repaired; for pigs, it is 7 mm [30]. Besides, due to limited tissue sources, ACC transplantation requires two operations, which is more traumatic and has unstable phenotype. In recent years, it has been found that the long-term efficacy of microfractures is not good. The better proliferation ability of juvenile cartilage has made it a more promising treatment plan for cartilage injuries. Although some PJACs have been applied to the repair of cartilage defects and have achieved certain clinical efficacy, whether the donor cells are involved in the process of repairing cartilage defect from the histological point of view remains unknown. To explore the repair mechanism of juvenile cartilage, we chose Guizhou minipigs as the large animal model to simulate the process of PJAC transplantation. Compared with dogs and other smaller animal models, the size, weight, and cartilage thickness of pigs are much closer to those of humans, and they are physiologically and physically more similar to us, and the cartilage defect repair process of pigs may also be closer to that of humans. Therefore, this study is of high clinical reference value.

This study also has limitations, such as the number of animal samples was relatively small; the observation period was limited to less than 3 months instead of a longer period of time; besides, due to limited funds, we did not use a blank control group and had not conducted further in-depth study to investigate the proliferation ability, etc. More long-term in-depth research will be conducted in the future.

## Conclusion

A large animal model was established with Guizhou minipigs, and the expression of Ki-67 protein and Lin28 protein in the repaired transplanted tissue was detected by immunohistochemistry. This large animal experiment has confirmed that the proliferation ability of PJAC within 3 months of transplantation was stronger than that of adult cartilage; it has studied the proliferation mechanism of PJAC, indicating that the enhanced expression of Lin28 was one of the mechanisms by which PJAC achieved stronger proliferation ability than adult cartilage; it has also explained the mechanism for the improvement of imaging data and clinical symptoms, which has become an important supplement to the clinical research of PJAC. PJAC technology has a good application prospect in repairing cartilage defects, but the related repair mechanisms still need to be further studied.

## Data Availability

The datasets used and analyzed during this study are available from the corresponding author on reasonable request, taking into account any confidentiality.

## Abbreviations

PJAC: Particulated juvenile allograft cartilage
ACC: Autologous cartilage chip transplantation
SEM: Standard error of mean
BSA: Bovine serum albumin
PBS: Phosphate buffer saline
DAB: Diaminobenzidine
IKDC: International Knee Documentation Committee
KOOS: Osteoarthritis Outcome Score
VAS: Visual analog scale
